# Six-month surveillance of *Candida parapsilosis* in Tyrol, Austria: high-risk ST11 lineage and early, heterogeneous fluconazole resistance

**DOI:** 10.3389/fcimb.2026.1771561

**Published:** 2026-03-30

**Authors:** Stephan Steixner, Roya Vahedi-Shahandashti, David Eisele, Werner Ruppitsch, Cornelia Lass-Flörl

**Affiliations:** European Excellence Centre of Medical Mycology (ECMM), Institute of Hygiene and Medical Microbiology, Medical University of Innsbruck, Innsbruck, Austria

**Keywords:** antifungal susceptibility testing, *Candida parapsilosis*, fluconazole resistance, outbreak investigation, whole-genome sequencing

## Abstract

**Introduction:**

*Candida parapsilosis* is an emerging cause of invasive candidiasis, driven by nosocomial transmission, environmental persistence, and rising azole resistance. Following an increase in detections at our institution, we investigated clinical *C. parapsilosis* isolates collected in Tyrol, Austria (February–July 2024).

**Methods:**

Susceptibility to fluconazole and anidulafungin was assessed by CLSI broth microdilution (BMD) and gradient diffusion (Etest) methods. Clonal relatedness was evaluated by whole-genome sequencing (WGS) with SNP-based phylogeny and multilocus sequence typing (MLST); azole-associated genes were analyzed in fluconazole non-susceptible isolates.

**Results:**

WGS demonstrated a genetically diverse population comprising six clusters, arguing against a single clonal outbreak. The most concerning cluster contained closely related ST11 isolates linked to previously described epidemic ST11 lineages and included both fluconazole-susceptible and non-susceptible isolates; non-susceptibility occurred in only one patient. Non-susceptible isolates carried multiple predominantly heterozygous non-synonymous variants across azole-associated pathways, consistent with an early, genetically heterogeneous stage of resistance development. Marked method-dependent discrepancies were observed for anidulafungin (all susceptible by CLSI BMD, but a substantial fraction non-susceptible by gradient diffusion method), while fluconazole showed higher concordance with minor misclassification.

**Discussion:**

Combining WGS with standardized susceptibility testing differentiated increased prevalence from clonal transmission, identified a high-risk ST11 lineage with a complex, likely polygenic resistance architecture, and highlighted limitations of gradient diffusion testing for echinocandin categorization. These findings support molecular surveillance and cautious interpretation of gradient diffusion susceptibility testing results to guide infection control and antifungal stewardship.

## 1 Introduction

The healthcare-associated burden of fungal infections continues to rise, significantly contributing to morbidity and mortality among hospitalized patients worldwide (Bongomin et al., 2017; Rayens and Norris, 2022; Cornely et al., 2025). Candidiasis is a common fungal infection that can manifest in diverse clinical forms and is a major contributor to the global burden of fungal diseases (Lass-Flörl et al., 2024; Cornely et al., 2025). While *C. albicans* remains the most prevalent species involved in candidiasis, a concerning global shift toward non-*albicans Candida* (NAC) species has been observed (Lamoth et al., 2018; Pappas et al., 2018). This shift is clinically important because of the potential for antifungal resistance and the associated risk of treatment failure (Whaley et al., 2016; Lass-Flörl et al., 2024).

Among these NAC, *C. parapsilosis* ranks as the second to third most prevalent species, with its occurrence varying by patient group, clinical setting, and geographic region (Toth et al., 2019). *C. parapsilosis* possesses several traits that confer a selective advantage for its persistence and prevalence in hospital settings, including high transmissibility and unique colonization patterns (Govrins and Lass-Flörl, 2024). It exhibits a strong capacity to attach and form persistent biofilms on indwelling catheters and implanted medical devices, posing a serious risk to patients undergoing invasive procedures (Toth et al., 2019; Daneshnia et al., 2023; Govrins and Lass-Flörl, 2024). *C. parapsilosis* infections primarily occur in patients admitted to intensive care units (ICUs) (Pinhati et al., 2016) and neonatal intensive care units (NICUs) (Hernandez-Castro et al., 2010; Qi et al., 2018), and associated outbreaks have been increasingly reported (Magobo et al., 2020; Thomaz et al., 2021). It is also one of the most common causes of candidemia in NICUs, particularly among neonates with risk factors such as low birth weight, indwelling catheters, parenteral nutrition, and mechanical ventilation (Harrington et al., 2017; Govrins and Lass-Flörl, 2024).

Fluconazole has long been the first-line therapy for *Candida* infections (Pappas et al., 2018). However, its effectiveness has been questioned due to the shift toward NAC species and the rising incidence of azole resistance, especially fluconazole resistance (Whaley et al., 2016; Berkow and Lockhart, 2017). This is primarily driven in *C. parapsilosis* primarily by *ERG11* point mutations affecting the azole target lanosterol 14α-demethylase (Berkow and Lockhart, 2017; Corzo-Leon et al., 2021), as well as by additional resistance mechanisms involving genes such as *MRR1*, *ERG3*, *UPC2*, *TAC1*, and *CDR1B* (Branco et al., 2023). Echinocandins are currently considered the first-line empirical therapy for candidiasis (Pappas et al., 2018; Cornely et al., 2025). However, *C. parapsilosis* naturally exhibits reduced *in vitro* susceptibility to echinocandins compared to other *Candida* species because of a P660A substitution in the Fks1 subunit of beta-glucan synthase (Garcia-Effron et al., 2008; Kontoyiannis et al., 2017). In addition, other acquired mutations in hot spot regions 1 and 2 of the *FKS* gene, such as R658S, L1376F, or F652S have been associated with echinocandin resistance (Siopi et al., 2022; Khalifa et al., 2024). *C. parapsilosis*, which has historically been linked to clonal outbreaks of drug-susceptible strains, has been associated with adult outbreaks caused by drug-resistant isolates since 2018 (Daneshnia et al., 2023), mostly fluconazole-resistant (Arastehfar et al., 2020; Fekkar et al., 2023). Notably, infections caused by fluconazole-resistant *C. parapsilosis ERG11*^Y132F^ mutants have been associated with a threefold increase in mortality compared to those with susceptible or non-susceptible strains without the mutation (50% *vs*. 16%) (Arastehfar et al., 2021). Therefore, the recognition of nosocomial outbreaks and the implementation of effective infection control measures require a rapid and reproducible method for distinguishing closely related isolates.

Following an increase in detections at our institution, we investigated clinical *C. parapsilosis* isolates collected in Tyrol, Austria (February–July 2024). This single-center study pursued three main objectives; first, to perform genetic analyses using whole-genome sequencing (WGS) to investigate the clonal nature and genetic relatedness of the isolates, second, to characterize the antifungal susceptibility profile of *C. parapsilosis* to fluconazole and anidulafungin, leading representative agents of the azole and echinocandin classes, using the CLSI broth microdilution (BMD) and the gradient diffusion (Etest) methods, and third, to determine the incidence of azole and echinocandin resistance during the study period and to investigate underlying resistance mechanisms in isolates exhibiting elevated minimum inhibitory concentrations (MICs).

## 2 Material and methods

### 2.1 Study design, sample collection, and fungal isolates

In this single-center study, all patient specimens that were culture-positive for *C. parapsilosis* and submitted to the Institute of Hygiene and Medical Microbiology at the Medical University Innsbruck from February 2024 to July 2024 were analyzed. Clinical specimens were received as part of routine diagnostics from different clinical institutions or general practitioners all over Tyrol, Austria, for bacterial and fungal infections and included tissue material, wound swabs, urine, puncture fluid, bronchoalveolar lavage fluid, feces, nail material, and one central venous catheter tip. No specific inclusion or exclusion criteria were applied to the study. Multiple isolates from the same patient were included to monitor changes in the antifungal susceptibility pattern and detect potential resistance-associated mutations.

After receipt of specimens, samples were analyzed according to local microbiological standards; cultural growth of *C. parapsilosis* was identified and confirmed by Matrix-assisted Laser Desorption Ionization Time of Flight (MALDI-TOF) mass spectrometry, as described by Steixner et al. (2025b), using a MALDI Biotyper smart (Bruker Daltonics, Bremen, Germany). As recommended by the manufacturer, a score of ≥2 was considered sufficient for species identification. After identification, the strains were stored at −20 °C and re-cultivated on Sabouraud’s dextrose agar (SDA) plates (BioMerieux, Vienna, Austria) at 37 °C for 24 h prior to the experiments.

### 2.2 Antifungal susceptibility testing and interpretation

Susceptibility testing for fluconazole and anidulafungin was performed by measuring the MIC according to yeast broth microdilution by CLSI BMD (CLSI, 2017), using fluconazole powder (Merck, Darmstadt, Germany) and anidulafungin powder (Merck, Darmstadt, Germany), and gradient diffusion method. *C. parapsilosis* ATCC 22019 and *C. krusei* ATCC 6258 were used as reference strains for all antifungal susceptibility tests. Gradient diffusion testing was performed on ready-to-use Roswell Park Memorial Institute (RPMI) 1640 agar plates (Axon-Lab, Tyrol, Austria) using fluconazole (BioMerieux, Vienna, Austria; 0.016 mg/L–256 mg/L) and anidulafungin (BioMerieux, Vienna, Austria; 0.002 mg/L–32 mg/L) Etest strips, as described previously (Vahedi-Shahandashti et al., 2025).

MIC values were obtained after 24 h of incubation by both methods and interpreted according to CLSI BMD breakpoints (CLSI, 2020) for both fluconazole and anidulafungin, categorizing isolates as susceptible (MIC ≤2 mg/L), intermediate (MIC of 4 mg/L), or resistant (MIC ≥8 mg/L). The MIC for both antifungals using the gradient diffusion method was determined at 80% inhibition, excluding any trailing observed, as described by the manufacturer. MICs were determined at 50% inhibition, as defined by CLSI (CLSI, 2017). For both antifungals, MIC_50_ and MIC_90_ values, defined as the MIC inhibiting the growth of 50% and 90% of isolates, respectively, were calculated.

### 2.3 DNA extraction

Genomic DNA of all isolates and the control strain *C. parapsilosis* ATCC 22019 (designated as CR6 in the present study) was extracted from 24 h-old SDA cultures using a cetyltrimethylammonium bromide (CTAB)-based method, adapted for fungal isolates with minor modifications, as described previously (Najafzadeh et al., 2010; Sun et al., 2010).

Briefly, fungal material was disrupted in TE (400 mM Tris, 10 mM Na-EDTA, pH 8.5–9) buffer with glass beads (G9143: 212–300 µm, Merck, Darmstadt, Germany), followed by 10% sodium dodecyl sulfate (Carl Roth, Karlsruhe, Germany) and proteinase K (Carl Roth, Karlsruhe, Germany) treatment, CTAB/NaCl precipitation, SEVAG (chloroform:isoamyl alcohol, 24:1) extraction, and isopropanol precipitation. Deviating from the aforementioned protocols, the samples were incubated at 65 °C instead of 55 °C, and the vortexing steps were extended to 5 min and 10 min. DNA was re-suspended in nuclease-free, DEPC-treated water (Carl Roth, Karlsruhe, Germany), and an RNase A digestion step with 2 µL RNase A (Qiagen, Hilden, Germany; 100 mg/mL) for 30 min at 37 °C, followed by 2 min at 65 °C, was performed prior to downstream applications.

### 2.4 Whole genome sequencing and sequence data analysis

Paired-end 2 × 150 bp whole-genome sequencing was performed at Eurofins Genomics (Eurofins, Constance, Germany) using an Illumina NovaSeq X+ (Illumina, San Diego CA, USA).

To detect single-nucleotide polymorphisms (SNPs) and structural variants, our WGS data, as well as data from three confirmed outbreaks, including outbreaks from Berlin (Brassington et al., 2025), Bloemfontein, and Johannesburg (Bergin et al., 2024), were analyzed using the perSVade pipeline v1.02.6 (Schikora-Tamarit and Gabaldon, 2022) with default settings and the recommended adjustments for diploid organisms. C*. parapsilosis* CDC317 was used as a reference. A comparison with known outbreak datasets was used as a reference to investigate the genetic relatedness of our isolates, allowing us to determine whether the isolates in our dataset showed levels of genetic similarity comparable to those observed in described clonal outbreaks. InterProScan v5.73-104.0 (Jones et al., 2014; Blum et al., 2025) was used to annotate the functional domains of genes carrying mutations.

A custom Python v3.11.8 (van Rossum, 2010) script was used to calculate the maximum pairwise symmetric SNP differences within the three known outbreaks (Bergin et al., 2024; Brassington et al., 2025) to assess the genetic relatedness of *C. parapsilosis* isolates and establish a suitable cluster cutoff for our dataset (https://github.com/DavidEiseleIMED/SymmetricSNP/tree/main). The libraries used within the Python script included the standard libraries re v2.2.1, csv v1.0, os, sys, and collections, and external libraries such as pandas v2.2.2 (McKinney, 2010; The pandas development team, 2024) and numpy v1.26.4 (Harris et al., 2020). From the homozygous SNPs that were called by all three SNP-callers employed by perSVade, such as bcftools (Li, 2011; Danecek et al., 2021), freebayes (Garrison and Marth, 2012), and HaplotypeCaller (Poplin et al., 2017; van der Auwera and O’Connor, 2020), a merged vcf file was constructed with bcftools merge v1.21. The script vcf2phylip (Edgardo M. Ortiz, 2018) was used to construct pseudogenomes, which were then analyzed using IQ-tree (Nguyen et al., 2015). Sequences with unusual GC content were analyzed using FastQ Screen to test for contamination (Wingett and Andrews, 2018). iTOL v7.4 was used for the visualization and annotation of the phylogenetic tree (Letunic and Bork, 2024). The phylogenetic tree was rooted using the “root on midpoint” option. In total, five pseudogenomes from our samples failed IQ-tree’s composition χ^2^-test, including CP18, CP21, CP22, CP43, and CP61. To identify genetic clusters, the maximum symmetric SNP difference was calculated for each of the three outbreaks investigated. The highest symmetric SNP difference of 731 SNPs was found in the Berlin outbreak and was used in the present study as a threshold for being considered at the same level of genetic similarity. The symmetric SNP difference within the Johannesburg outbreak was the lowest at 310, and the Bloemfontein outbreak with 557 was intermediate.

### 2.5 Multilocus sequence typing

The sequences of our isolates, as well as those of the three known outbreaks from Berlin (Brassington et al., 2025), Bloemfontein, and Johannesburg (Bergin et al., 2024), were analyzed using a multilocus sequence typing (MLST) annotation tool developed by Seemann (2018) v2.23.0 to which the MLST scheme by Brassington et al. (2025) was added. To avoid IUPAC ambiguities, as recommended by Seemann’s MLST annotation tool (Seemann, 2018), the alternative allele was used to determine the sequence type (ST) when a heterozygous mutation was present. STs for both the reference and alternative alleles are provided in the **Supplementary Material S1**. Novel sequence types not covered by the original MLST scheme were added to the scheme shown in **Supplementary Material S2**.

### 2.6 Statistical analysis

All statistical analyses were performed using GraphPad PRISM v10.2.3. To evaluate the changes in the number of *C. parapsilosis* isolates from February to July in 2022, 2023, 2024, and 2025, monthly data were analyzed at the isolate, patient, and isolate-per-patient (IPP) levels. IPP ratios were calculated by dividing the total number of isolates by the total number of patients. Data normality was assessed using the Shapiro–Wilk test, and homogeneity of variance across groups was evaluated using the Brown–Forsythe test. Differences between years were analyzed using one-way analysis of variance (ANOVA), followed by the Holm–Šídák multiple-comparison test. Susceptibility testing results were compared using the Wilcoxon matched-pair signed-rank test. Statistical significance was set at p <0.05.

## 3 Results

### 3.1 Increasing trend of *C. parapsilosis* isolation compared to previous years

Between February and July 2024, *C. parapsilosis* was cultured from 47 clinical specimens collected from 24 patients at our institution, resulting in an IPP ratio of 1.96. The isolates originated from various body sites (**Supplementary Table 1**). Multiple isolates were obtained from some patients as part of routine clinical care, either from different anatomical sites or through repeated sampling over time. The anatomical sites and dates of collection for each isolate are provided in **Supplementary Table 1**. During the corresponding period, the IPP ratio increased from 1.22 in 2022 (22 isolates from 18 patients) and 1.48 in 2023 (43 isolates from 29 patients) to 1.96 in 2024 (47 isolates from 24 patients), indicating an upward trend in IPP. Recurrent isolates from the same patient were retained for further analysis to assess potential changes in antifungal susceptibility patterns and investigate the occurrence of resistance-associated mutations over time.

### 3.2 Method-dependent differences in antifungal susceptibility testing profile

Fluconazole gradient diffusion testing resulted in a median MIC of 1 mg/L (range: 0.125 mg/L–≥32 mg/L), whereas CLSI BMD yielded a median MIC of 0.5 mg/L (range: 0.125 mg/L–4 mg/L). Two isolates (obtained from one patient) were classified as resistant by the gradient diffusion method but were considered intermediate by CLSI BMD. For anidulafungin, the median MICs were 2 mg/L using the gradient diffusion method and 1 mg/L using CLSI BMD. MIC ranges differed considerably between the methods: 0.004 mg/L–16 mg/L for Etest versus 0.016 mg/L–2 mg/L for CLSI BMD. Gradient diffusion testing classified three isolates (obtained from two patients) as resistant and eight isolates (obtained from two patients) as intermediate, whereas CLSI BMD categorized all isolates as susceptible.

**Figure 1:**
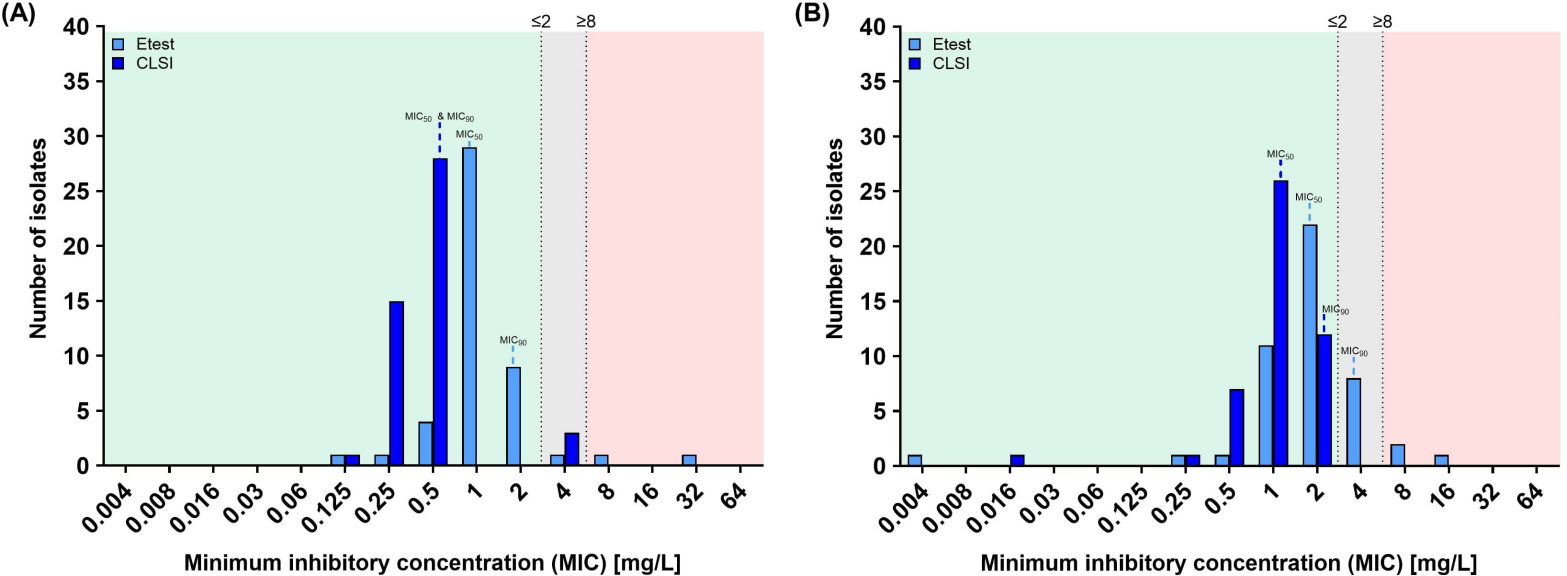
Minimum inhibitory concentration (MIC) distribution of **(A)** fluconazole and **(B)** anidulafungin from *Candida parapsilosis* isolates (n = 47) using gradient diffusion (Etest) and broth microdilution (CLSI) methods. Multiple isolates from the same patient are included; therefore, MIC distributions reflect isolate-level rather than patient-level frequency. Clinical and Laboratory Standards Institute (CLSI) breakpoints (CLSI, 2020) are visually indicated by background shading and lines: green for susceptible (MIC ≤2 mg/L), gray for intermediate (MIC = 4 mg/L), and red for resistant (MIC ≥8 mg/L) categories. Gradient diffusion obtained MICs were rounded to the next higher log2 dilution for comparison reasons.

Overall, fluconazole antifungal susceptibility testing (AFST) by both methods classified 94.1% of isolates as susceptible (**Figure 1A**). Gradient diffusion and CLSI BMD classified 2.1% and 6.4% of strains as intermediate, respectively, while only gradient diffusion testing classified 4.3% of isolates as resistant, whereas CLSI BMD did not classify any strain as resistant. For anidulafungin (**Figure 1B**), all isolates were classified as susceptible by CLSI BMD, whereas gradient diffusion testing classified 76.6% of the isolates as susceptible, 17.0% as intermediate, and 6.4% as resistant. As repeated isolations from the same patient were analyzed, the MIC distributions should be interpreted as isolate-level frequencies and not as patient-level prevalence.

Statistical analysis revealed a significant method-dependent difference in the categorization of both antifungals tested (p <0.0001). Essential agreement, defined as MIC values within ± one two-fold dilution step between gradient diffusion and CLSI BMD, was observed in 55.3% of cases for fluconazole and 74.5% for anidulafungin. Discrepancies were further classified into very major errors (VME, gradient diffusion-susceptible/CLSI BMD-resistant), major errors (ME, gradient diffusion-resistant/CLSI BMD-susceptible), and minor errors (MiE, one intermediate method, the other susceptible or resistant). No VMEs were observed for either antifungal agent. However, three (6.4%) ME were detected for anidulafungin. MiEs were notably more frequent for anidulafungin (17.0%) than for fluconazole (4.3%), indicating a greater categorical discrepancy between the two testing methods for anidulafungin.

### 3.3 Cluster investigation and MLST classification

**Figure 2:**
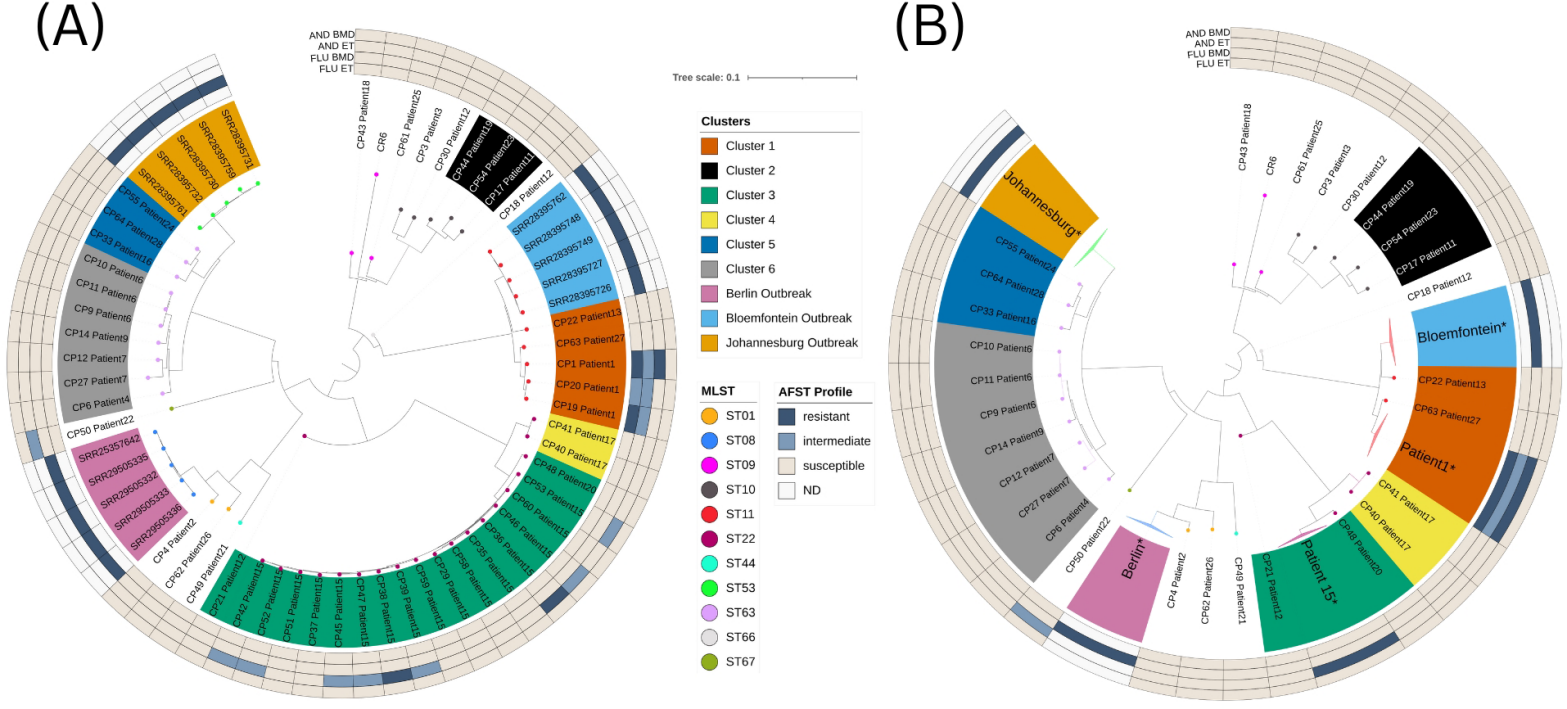
Phylogram based on homozygous single-nucleotide polymorphisms (SNPs) of *Candida parapsilosis* isolates from this study (n = 47) and a reference strain (*C. parapsilosis* ATCC 22019; CR6) together with isolates from three previously described outbreaks: Berlin (n = 38) (Brassington et al., 2025), Bloemfontein (n = 14), and Johannesburg (n = 12) (Bergin et al., 2024). Phylogeny is shown at **(A)** isolate-level and **(B)** patient-level. The phylogeny created by IQ-tree, which was based on the pseudogenomes of around 11,000 homozygous variable positions, was visualized using iTOL. A tree distance of 0.1 corresponds to around 1,000 homozygous SNP differences. The isolates from the three different outbreaks were condensed to the most related five isolates for each of the outbreaks. As a threshold, 731 SNPs, based on the already described, uncondensed outbreaks (Bergin et al., 2024; Brassington et al., 2025) of *C. parapsilosis*, were used to determine clusters. Sequence types (ST) were determined by multilocus sequence typing (MLST) analysis (Seemann, 2018; Brassington et al., 2025), and are highlighted in colored dots. The different clusters are shown by the colored label backgrounds. Susceptibility profile of fluconazole and anidulafungin against isolates according to gradient diffusion method (using Etest strips) and broth microdilution (Clinical Laboratory Standards Institute (CLSI)) are shown in beige (susceptible), light-blue (intermediate) and dark-blue (resistant) according to CLSI breakpoints (CLSI, 2020). All antifungal susceptibility testing (AFST) results were acquired using CLSI except the results from the Berlin outbreak which were performed according to EUCAST. In **(B)**, entries marked with an asterisk (*) represent multiple isolates condensed for visualization; no isolates present in **(A)** were removed from the analysis. Further, the AFST with the highest minimum inhibitory concentration (MIC) results are shown for these entries. AND, anidulafungin; FLU, fluconazole; BMD, broth microdilution; ET, Etest gradient diffusion; ND, not determined.

MLST and SNP analyses showed no clonal outbreak but a diverse *C. parapsilosis* population. Our isolates belonged to nine STs (ST01, two isolates obtained from two patients; ST09, 2/2; ST10, 5/5; ST11, 5/3; ST22, 20/4; ST44, 1/1; ST63, 10/7; ST66, 1/1; and ST67, 1/1), six SNP clusters, including one pseudocluster (cluster 4), and 10 singletons (**Table 1**, **Figure 2**).

**Table 1:** Internal symmetric SNP difference.

Cluster	Minimum SNP difference	Maximum SNP difference	Sequence type
Cluster 1	65	499	ST11
Cluster 2	524	539	ST10
Cluster 3	35	261	ST22
Cluster 4*	51	51	ST22
Cluster 5	185	362	ST63
Cluster 6	61	448	ST63
Bloemfontein	124	537	ST11
Johannesburg	140	310	ST53
Berlin	28	731	ST01, ST08, ST68

^*^Cluster 4 can be regarded as a pseudocluster.

SNP, Single nucleotide polymorphism.

Symmetric SNP differences of clusters, outbreaks, and singletons were similar but above the defined cluster threshold (**Table 2**).

**Table 2:** Symmetric SNP differences between clusters, outbreaks and singletons.

Cluster		Minimum SNP difference	Maximum SNP difference
Cluster 1	Bloemfontein	899	1,283
Cluster 3	Cluster 4*	808	839
Cluster 5	Cluster 6	762	852
CP4	Berlin	557	744
CP62	Berlin	853	999
CP4	CP62	758	758
CP3	Cluster 2	1,195	1,242
CP30	Cluster 2	1,111	1,160

^*^Cluster 4 can be regarded as a pseudocluster.

SNP, Single nucleotide polymorphism.

Cluster 1 isolates derived from three different patients, with patient 1 with three isolates (CP1, CP19, CP20) and patients 13 and 27 with isolates CP22, and CP63, respectively, which differs from the ST11 Bloemfontein outbreak isolates by a minimum of 899 SNPs (**Table 2**). ST10 was present in three isolates/patients of Cluster 2 (patients 11, 19, and 23; CP17, CP44, and CP54, respectively) and the singletons CP3 (patient 3) and CP30 (patient 12). ST09 was found in two singletons/patients (patients 18 and 25; CP43 and CP61, respectively) and the control strain (CR6). All isolates/patients from Clusters 3 and 4 belonged to ST22. The newly described ST63 included three isolates/patients from Cluster 5 (patients 16, 24, and 28; CP33, CP55, and CP64, respectively) and seven isolates/four patients from Cluster 6 (patients 4, 6, 7, and 9; CP6, CP9-11, CP12 and CP27, and CP14, respectively). The singleton/patient CP4 (patient 2) was genetically related to the Berlin outbreak. No patient with isolates from different clusters was found. Patient 12 had isolates belonging to three different STs, namely ST22 (CP21) from cluster 3, and two singletons, ST66 (CP18) and ST10 (CP30).

Some isolates from cluster 1, all from the same patient, showed decreased susceptibility or resistance: isolates CP1 and CP19 showed resistance against fluconazole by the gradient diffusion method, were classified as intermediate by CLSI BMD, and isolate CP20 was categorized as fluconazole-intermediate by both methods. Nine isolates from patient 15 in Cluster 3 showed alterations in AFST by gradient diffusion testing for anidulafungin: two isolates (CP36 and CP38) showed anidulafungin resistance, while seven isolates (CP39, CP42, CP45, CP46, CP47, CP52, and CP53) were classified as intermediate to anidulafungin.

### 3.4 Azole-resistance-associated mutations identified in non-susceptible isolates from a single patient

The three fluconazole non-susceptible isolates (CP1, CP19, and CP20; Cluster 1), all originating from the same patient and categorized as intermediate by CLSI BMD and intermediate/resistant by gradient diffusion testing, were analyzed for exclusive SNPs shared between them and absent in all susceptible isolates to identify SNPs potentially responsible for their decreased susceptibility (intermediate to fluconazole). A total of 39 genes with nonsynonymous SNPs were discovered (**Table 3**), of which 26 were SNPs within functional domains recognized by InterProScan. Of these mutations, three were homozygous and the other 23 were heterozygous. Of the remaining 13 mutations detected outside the InterPro domains, three were homozygous and 10 were heterozygous. CP1, CP19, and CP20 also did not carry the *ERG11*^Y132F^ mutation present in the reference genome or the *MRR1*^A854V^ mutation detected in the Bloemfontein outbreak (Bergin et al., 2024); instead, they showed the same genotype as susceptible strains.

**Table 3:** Mutation analysis of non-synonymous single nucleotide polymorphisms exclusively present in strains with lower susceptibility to fluconazole.

Gene ID	Protein name (similar to)^#^	Protein function of similar protein^###^	Mutation
Inside InterPro domain			
XM_036806891.1	XP_036662873.1 (FLO8^##^)	Cell–cell-adhesion, flocculation, invasive growth	S260L
XM_036807146.1	XP_036662896.1 (SEC27)	ER-to-Golgi vesicle-mediated transport, localization of intracellular mRNAs	A778V
XM_036807519.1	XP_036664348.1 (DFI1)	Calcium-mediated signaling, cell adhesion, invasive filamentous growth, MAPK cascade	N347D
XM_036807888.1	XP_036664680.1 (RRD1)	Autophagy, stress-response, DNA repair, G1/S transition of mitotic cycle, mitotic spindle organization	L46Q
XM_036807900.1	XP_036664691.1 (HDA1)	Filamentous growth, cellular response to pH and starvation, histone deacetylase	E654*
XM_036807998.1	XP_036664778.1 (MTC5)	TORC1 regulation, protein transport, cellular response to amino acid starvation	A200P
XM_036808069.1	XP_036664842.1 (ATP25)	Stability of ATP9 mRNA, assembly of F1F0 ATP synthase complex	E272K
XM_036808075.1	XP_036664848.1	**–**	**G112R**
XM_036808173.1	XP_036664936.1 (FTH2)	Iron ion transmembrane transport, prostaglandin metabolic process	E173K
XM_036808310.1	XP_036665060.1 (DJP1)	Peroxisome organization	R27*
XM_036808538.1	XP_036665265.1 (Hyphally regulated cell wall protein N-terminal family protein	–	P132R
XM_036809930.1	XP_036666518.1 (SHQ1)	Unfolded protein binding, box H/ACA snoRNP assembly	M271V
XM_036810024.1	XP_036666602.1 (TPP1/PNK1)	DNA repair	V106I
XM_036810053.1	XP_036666629.1 (TBF1)	Negative regulation of chromatin silencing and telomere maintenance	S396N
XM_036810192.1	XP_036666754.1 (ABZ2)	Carboxylic acid biosynthesis	H81R
XM_036810736.1	XP_036667243.1 (FOX2)	Fatty acid beta-oxidation	K321E
XM_036811024.1	XP_036667502.1 (ARH1)	Ubiquinone biosynthesis, iron homeostasis	R9*
XM_036811176.1	XP_036667640.1 (RCH1)	Cytosolic calcium homeostasis	P216T
XM_036811287.1	XP_036667740.1 (TRM3)	tRNA methylase	H59Y
XM_036811323.1	XP_036662822.1 (FIG4)	Phosphatidylinositol phosphate catabolism	G496S
XM_036811675.1	XP_036668088.1 (JEN1)	Transport of selenite, pyruvate and lactate	**A232E**
XM_036811700.1	XP_036668111.1 (BLM3)	DNA repair	**T856I**
XM_036811751.1	XP_036668157.1	–	L355S
XM_036812158.1	XP_036668523.1 (ENP2)	18S rRNA and ribosomal subunit biogenesis	M35I
XM_036812236.1	XP_036663364.1	–	A278V
XM_036812375.1	XP_036663489.1(HEM13)	Heme biosynthesis	H127R
Outside InterPro domain			
XM_036806997.1	XP_036663877.1	**–**	**S374N**
XM_036807790.1	XP_036662954.1	–	L543F
XM_036807853.1	XP_036664649.1 (LRG1)	1,3-β-glucan biosynthesis, cell wall organization	G1284E
XM_036807904.1	XP_036664694.1	–	F10L
XM_036808000.1	XP_036664780.1(NdufS8)	NADH-quinone oxidoreductase	L47S
XM_036808083.1	XP_036664855.1 (ATG4)	Proteolysis, macroautophagy, cytoplasm-to-vacuole targeting pathway, late nucleophagy, piecemeal microautophagy of nucleus, protein lipidation, mitochondrion degradiation	L409M
XM_036808735.1	XP_036665442.1	–	D138G
XM_036809702.1	XP_036666312.1	–	S109N
XM_036810417.1	XP_036666956.1 (ZRT1, nimA)	Zinc ion transport, serine/threonine kinase, filamentous growth	**P161R**
XM_036811088.1	XP_036667561.1 (PGA30)	Ribosomal small subunit biogenesis	A251S
XM_036811551.1	XP_036667976.1 (MSC7)	Reciprocal meiotic recombination	**A7T**
XM_036811764.1	XP_036668169.1 (SUC1 transcriptional regulator; GAL4-like)	Sucrose catabolism	V402S
XM_036812622.1	XP_036662866.1	–	T38I

^#^nucleotide/protein sequences named in brackets are sequences with the highest similarity according to BLASTn and BLASTp (query coverage >90%, percentage identity >70).

^##^low similarity (67%).

^###^Information on protein function has been extracted from uniprot.org and yeastgenome.org.

Mutations mentioned in bold are considered homozygous.

ER, endoplasmic reticulum.

In addition to SNPs, two structural variant mutations were found to be unique to CP1, CP19, and CP20, which showed lower susceptibility to fluconazole. An in-frame deletion in an uncharacterized gene (XM_036809809.1) reaching from protein positions 20–27 (DNNNNNNN/D) was shared between all three isolates. The mutation was located inside an Ipl1-aurora-like kinase (IAL)-related serine/threonine protein kinase domain (PTHR24350), as identified by InterProScan. Additionally, another uncharacterized gene (XM_036808562.1) showed duplication outside an InterPro domain.

## 4 Discussion

The rising prevalence, environmental persistence, and growing azole resistance have made *C. parapsilosis* a global health threat (Pinhati et al., 2016; Bergin et al., 2024; Brassington et al., 2025). Its capacity for nosocomial transmission (Escribano and Guinea, 2022; Daneshnia et al., 2023; Wijaya et al., 2023; Steixner et al., 2025a) further increases its clinical impact, similar to that of *Candidozyma auris* (formerly *Candida auris*). The emergence of fluconazole-resistant lineages presents a significant infection control challenge that calls for careful monitoring and molecular typing (Semet et al., 2025; Steixner et al., 2025a; Vena et al., 2025). A recent increase in the recovery of *C. parapsilosis* from multiple patients over time at our institution prompted a combined molecular and phenotypic investigation to clarify genetic relatedness, determine antifungal susceptibility profiles, and investigate potential resistance mechanisms.

Whole-genome sequencing revealed six genomic clusters but no evidence of a single, ongoing clonal outbreak. Furthermore, no patient harbored isolates belonging to more than one genetic cluster at any time point. Notably, three isolates (CP18, CP21, and CP30) recovered from patient 12 from the same anatomical site each belonged to a distinct sequence type (ST66, ST22, and ST10, respectively), a pattern that suggests the presence of multiple genetically unrelated strains within the same patient, which is more consistent with independent infection or colonization events than a single strain diversification. The most concerning cluster (Cluster 1) comprised closely related ST11 isolates in three patients, including three fluconazole-non-susceptible strains from patient 1 (CP1, CP19, and CP20) and one susceptible strain from patients 13 and 27 (CP22 and CP63) that were genetically linked to previously described epidemic ST11 lineages from the Bloemfontein outbreak (Bergin et al., 2024). The *MRR1*^A854V^ mutation, which was described in eight of the 14 strains from the Bloemfontein outbreak, was not detected in our isolates. Likewise, the *ERG11*^Y132F^ mutation, published in the Johannesburg (Bergin et al., 2024) and Berlin outbreaks (Brassington et al., 2025), was also not detected in our isolates, suggesting that these common azole resistance mechanisms were not involved in our study. Since fluconazole non-susceptibility remained confined to isolates from a single patient, our data are more consistent with the repeated recovery of a high-risk lineage across three patients rather than a large clonal outbreak. This interpretation should be considered in the context of the retrospective, single-center, laboratory-based design of this study. Nevertheless, the close relatedness of our isolates to known epidemic strains, even though observed in only a limited number of patients, underlines the need for ongoing molecular surveillance and careful antifungal stewardship (Steixner et al., 2025a). Future studies incorporating extended surveillance periods, larger patient cohorts, and detailed clinical metadata, including ward location and hospitalization history, are required to monitor and confirm the potential expansion of ST11 and its associated resistance mechanisms.

Mutational analysis of the three fluconazole-non-susceptible isolates from one patient (patient 1; CP1, CP19, and CP20) identified multiple non-synonymous SNPs that were absent from all susceptible strains (**Table 3**). Several affected genes have been implicated in azole response or resistance in *Candida* spp., including *HEM13*, which carries a mutation in the oxygen-dependent coproporphyrinogen-III oxidase domain. It contributes to the biosynthesis of heme, a prosthetic group of several ergosterol biosynthesis enzymes, including ERG11. Increased *HEM13* expression of has been suggested to contribute to increased resistance to fluconazole (Pais et al., 2020). Furthermore, a mutation in the Na^+^/H^+^ antiporter-like fold domain of *RCH1*, linked to Ca^2+^ homeostasis and signaling, was found. It has been demonstrated, that *RCH1* deletion leads to increased azole tolerance in *C. albicans*, which is speculated to be related to Ca^2+^ homeostasis (Jiang et al., 2012). This is supported by previous studies showing that deletion of a different Ca^2+^ transporter (PMC1) and its influence on calcineurin signaling are associated with azole tolerance (Cruz et al., 2002; Edlind et al., 2002; Sanglard et al., 2003). A mutation in the WD repeat domain of *MTC5*, a regulator of TORC-1 signaling, was found (Dokudovskaya and Rout, 2015). An overactive TORC-1 pathway increases azole resistance in *C. albicans* by activating Hsp90 and stabilizing calcineurin (Khandelwal et al., 2018; Miao et al., 2025). We also identified a mutation in the functional domain of the iron transporter gene *FTH2*. This gene has been linked to increased azole resistance, although the underlying mechanism remains unknown (van Genechten et al., 2024). Another mutation found in our study introduced a stop codon into the Arb2-like domain of the histone deacetylase gene, *HDA1*. In contrast to our AFST results, previous research found that deletion of *HDA*1 leads to reduced expression of efflux pumps (*CDR1*, *CDR2*, *MDR1*, and *FLU1)* and thus to increased susceptibility to azoles (Li et al., 2015). We also identified a mutation in the transcription activator domain of a *FLO8*-like transcription factor. Deletion of *FLO8* has been shown to confers azole resistance by upregulating the efflux pumps CDR1 and CDR2 (Li et al., 2019). A mutation in *NdufS8*, an Fe–S cluster protein of respiratory complex I, may also be relevant, as the inactivation of respiratory complex I has been shown to increase azole resistance, potentially by reducing oxidative stress (Bromley et al., 2016). Additionally, other Fe–S cluster proteins have been reported to influence azole resistance. The plasma membrane zinc transporter *ZRT1*, which has been observed to be downregulated in fluconazole-resistant *C. glabrata* (Caudle, 2010), also carried a mutation.

Notably, most of these variants were heterozygous. The co-existence of susceptible and fluconazole-non-susceptible isolates within a single ST11 cluster, together with the predominance of heterozygous variants affecting multiple azole-associated pathways and the method-dependent shift from intermediate to resistant MIC categories, suggests an early and genetically heterogeneous stage of resistance development rather than a fully fixed resistant genotype. Although heteroresistance assays were not performed in our study (Knoll et al., 2022), our findings are consistent with a mosaic-like resistance architecture within this high-risk lineage and warrant further functional investigation.

In addition, we compared two commonly used AFST approaches: the gradient diffusion method and BMD. While the gradient diffusion method is widely applied in routine diagnostics because it is simple to perform, less labor-intensive, and provides rapid results, BMD represents the gold standard reference method for AFST (Berkow et al., 2020). Given the extensive use of the gradient diffusion method in clinical practice, evaluating its performance relative to the reference method is of practical relevance. Previous studies have reported discrepancies between the gradient diffusion method and CLSI BMD results (Serrano et al., 2003; Arendrup et al., 2010; Dannaoui et al., 2010; Vahedi-Shahandashti et al., 2025). Such differences may lead to misclassification of susceptibility categories and, consequently, may influence clinical decision-making and antifungal therapy. Therefore, comparing the gradient diffusion method with CLSI BMD in this study was intended to assess the level of agreement between routine diagnostic practice and the reference standard.

In our cohort, anidulafungin results showed pronounced method-dependent discrepancies, as all isolates were categorized as susceptible by CLSI BMD, but gradient diffusion testing classified nearly one-quarter as intermediate or resistant, resulting in substantial essential disagreement and misclassification. These findings indicate that anidulafungin gradient diffusion results may overestimate non-susceptibility and should be interpreted cautiously, particularly when used to guide echinocandin therapy decisions. For fluconazole, the discrepancies between the two methods were less marked but still clinically relevant. CLSI BMD identified three intermediate isolates, two of which were categorized as resistant by the gradient diffusion method, and the overall essential agreement was modest. Although only a small proportion of isolates were misclassified, fluconazole serves as a class representative for azoles, and even minor inconsistencies may distort local resistance surveillance and influence treatment decisions. Therefore, our data support the continued validation of gradient diffusion methods for azole testing and highlight the importance of confirming critical results using reference microdilution.

The present study has several limitations. First, this was a retrospective, single-center laboratory study conducted over a limited period (February–July 2024) with a modest sample size (47 specimens from 24 patients) and heterogeneous specimen types. In addition, repeated sampling from the same patient may have inflated isolate-level frequencies compared to patient-level estimates. Second, genomic clustering was based on an SNP distance threshold (731 SNPs) derived from previously published outbreak datasets. Although the analytical steps are transparent, the use of alternative clustering thresholds or additional sensitivity analyses may influence cluster assignment. However, all genomes fulfilled the basic quality criteria, including an average sequencing coverage >70×, an average unmapped reads percentage <1% (max 7%), appropriate genome size, and expected G+C content; nevertheless, some pseudogenomes failed the IQ-TREE composition χ² test, which should be considered when interpreting the phylogenetic analysis results. Third, the analyses of resistance mechanisms were limited to three fluconazole nonsusceptible isolates obtained from a single patient. Therefore, the identified genetic variants should be interpreted as hypothesis-generating, rather than as definitive drivers of resistance.

Taken together, our findings illustrate how the integration of WGS and standardized AFST can disentangle increased prevalence from clonal spread, detect emerging high-risk lineages, and provide first insights into the complex, polygenic nature of fluconazole non-susceptibility in *C. parapsilosis*. Simultaneously, they underscore the limitations of relying on a single susceptibility testing method. Future studies with larger cohorts, detailed clinical metadata and functional validation of candidate resistance mutations are essential to better understand the dynamics of resistance evolution in this species and to translate these insights into effective infection control and treatment strategies.

## 5 Conclusion

In summary, we observed an increased IPP prevalence of *C. parapsilosis* at our institution compared with previous years, driven by a genetically diverse population that included a high-risk ST11 lineage detected in three patients and related to previously described epidemic strains but without evidence of a single large clonal outbreak. By integrating WGS with AFST, we were able to unravel the increased prevalence of transmission events and link fluconazole non-susceptibility to a complex, predominantly heterozygous resistance architecture. Notably, pronounced method-dependent discrepancies, particularly for anidulafungin, suggest that the gradient diffusion method may overestimate non-susceptibility and should be interpreted cautiously, with critical results confirmed by reference microdilution. Together, these findings underline the importance of molecular surveillance and robust susceptibility testing for the early recognition of emerging high-risk *C. parapsilosis* lineages and for guiding antifungal stewardship. Future multicenter studies with larger cohorts, detailed clinical data, and functional validation of candidate mutations are needed to better define the dynamics and clinical impact of resistance evolution in this species group.

## Data Availability

The datasets generated and analyzed in this study can be found in the Sequence Read Archive (SRA) under the bioproject accession number PRJNA1348317. The custom Python script was uploaded to GitHub (https://github.com/DavidEiseleIMED/SymmetricSNP/tree/main).
